# Identifying quality of life domains and facets affected in relapsing polychondritis: a qualitative analysis for the development of a disease-specific health-related quality of life instrument

**DOI:** 10.1186/s13023-026-04297-3

**Published:** 2026-03-11

**Authors:** Philippe Mertz, Oliver Sander, Raquel Faria, Francesca Crisafulli, Sofia Silva-Ribeiro, Lou Kawka, Cedric Sztejkowski, Christina Düsing, Thomas Rose, Antonio Lamas, Carlos Vasconcelos, Giulia Fontana, Paolo Semeraro, Teodora Neagu, Mihaela Resteu, Laura Damian, Cristina Pamfil, Camelia Bucsa, Lisa J. Matthews, Hervé Devilliers, Simona Rednic, Laurent Arnaud

**Affiliations:** 1 Sorbonne université, Hôpital Tenon, DMU3ID APHP, ERN RITA, Paris, France; 2 Centre de référence des maladies auto-inflammatoires et de l’amylose inflammatoire (CEREMAIA), Paris, France; 3 Centre hospitalier de Versailles, le Chesnay, 78150 France; 4 Department for Rheumatology and Hiller Research Centre for Rheumatology, University Hospital, Düsseldorf, Germany; 5 Unidade de Imunologia Clínica, Hospital de Santo António, Unidade Local de Saúde Santo António, Porto, Portugal; 6 Rheumatology and Clinical Immunology Unit, ASST Spedali Civili of Brescia, Department of Clinical and Experimental Sciences, University of Brescia, Brescia, Italy; 7 Instituto Universitário de Lisboa, Lisboa, Portugal; 8 Department of Rheumatology, National Reference Center for Rare Autoimmune Diseases (RESO), Hôpitaux Universitaires de Strasbourg, Strasbourg, France; 9 Klinik für Rheumatologie und Klinische Immunologie, Charité Universitätsmedizin Berlin, Charité Platz 1, 10117 Berlin, Germany; 10 Department of Rheumatology, Emergency County Teaching Hospital, University of Medicine and Pharmacy Iuliu Hatieganu, Cluj-Napoca, Romania; 11 ASPOR Association of Romanian Relapsing Polychondritis Patients & Iuliu Hațieganu University of Medicine and Pharmacy, Cluj-Napoca, Romania; 12 Relapsing Polychondritis Awareness & Support, (relapsingpolychondritis.org), Worcester, UK; 13 Internal Medicine and Systemic Diseases Unit, University Hospital Centre Dijon, Dijon, France

**Keywords:** Relapsing polychondritis, Quality of life, Burden of disease

## Abstract

**Introduction:**

Relapsing polychondritis (RP) is a rare inflammatory disease characterized by recurrent inflammation of cartilaginous structures and a broad spectrum of systemic features. To date, no Health-related Quality of life (QoL) data are available in RP, and generic measures may not fully capture the unique burden faced by patients, given the specific nature of RP symptoms. This study aimed to identify QoL facets relevant for further inclusion in a disease-specific QoL instrument, the RP-QoL.

**Methods:**

An international study was conducted within the framework of the European Reference Network ReCONNET, using an online interview script designed by nine physicians, a clinical psychologist, and two patient representatives. The survey was cross-culturally adapted into five languages. Thematic analysis was performed by native speakers of each language and further aggregated to characterize QoL in RP.

**Results:**

A total of 274 patients (median age: 54yo, 86% women) from 22 countries completed the interview. The most affected QoL facets were ‘personal relationships’ (58.8%), ‘work capacity’ (51.8%), and ‘daily activities’ (47.8%). Additional concerns included mainly fatigue, pain, cognition, mental health and social life. A total of 67 patients (24.5%) identified 20 specific issues not fully captured by generic QoL instruments, such as difficulties for planning, verbal communication, vision impairment, as well as not being considered seriously by those around them.

**Conclusion:**

This study highlights the profound burden of RP and emphasizes the need for the development of a disease-specific health-related QoL instrument, as a way to improve patient-centered care and disease management.

**Supplementary Information:**

The online version contains supplementary material available at 10.1186/s13023-026-04297-3.

## Abstract (significance)

What is already known on this topic

What this study adds

How this study might affect research, practice, or policy

Relapsing polychondritis (RP) is a rare, inflammatory disease primarily affecting cartilaginous structures, and it presents with diverse systemic symptoms. However, no health-related quality of life (QoL) data exist specifically for RP, and generic QoL measures may fail to fully capture the unique challenges faced by these patients.

This study identifies specific QoL facets that are most impacted by RP, such as personal relationships, work capacity, and daily activities. It also highlights additional concerns like fatigue, pain, and mental health, which may not be adequately addressed by generic QoL instruments.

The findings of this study may guide the development of more tailored, patient-centered care for RP, leading to better disease management.

**Supplementary Information:**

The online version contains supplementary material available at 10.1186/s13023-026-04297-3.

## Introduction

Relapsing polychondritis (RP) is a rare disease characterized by recurrent inflammation of cartilaginous structures, including the ears, nose, joints, and respiratory tract [1]. RP may lead to severe and potentially life-threatening complications, such as airway collapse with acute respiratory failure [2]. Also, the chronic and relapsing nature of the disease, combined with the potential for damage, highlights the importance of assessing the Health-related Quality of life (HR-QoL) in affected individuals.

Relapsing polychondritis is a very rare disease, with an estimated prevalence ranging from 4.5 to 25 cases per million and limited epidemiological data, mostly from Europe [1, 3–5]. Underdiagnosis and diagnostic coding inaccuracies likely contribute to uncertainty in reported rates. The disease typically affects middle-aged adults but can occur at any age. Unlike other autoimmune diseases such as rheumatoid arthritis for which validated QoL tools exist (e.g., Rheumatoid Arthritis Quality of Life Questionnaire [6] or the Rheumatoid Arthritis Quality of life Scale [7]), RP lacks a disease-specific instrument. Moreover, RP encompasses very specific manifestations in comparison to other autoimmune diseases (e.g. chondritis, sensory organs involvement) that are not correctly assessed with generic QoL tools.

During the last decades, HR-QoL has emerged as a critical outcome measure in the management of chronic inflammatory and autoimmune diseases. HR-QoL refers to an individual’s perception of their living conditions in relationship with the disease, and is influenced by cultural values, personal expectations, and standards of living. It is a concept that encompasses not just physical health, but also emotional, social, and psychological well-being [8]. With the evolution of healthcare, the medical field has increasingly adopted a holistic approach, recognizing the importance of HRQoL in understanding the full impact of disease on a patient’s life, in addition to disease activity [9] and damage [10].

However, studies of HR-QoL are inexistent and there is to date no disease-specific instrument to assess HR-QoL in RP. Importantly, generic HR-QoL measures, while useful, may not adequately capture the specific challenges and concerns faced by RP patients. To this end, the European Reference Network on Connective Tissue and Musculoskeletal Diseases (ERN ReCONNET) aims to develop a disease-specific QoL assessment tool, the RP-QoL. Here, we present the qualitative study conducted to identify disease-specific QoL facets that will be used to develop the final RP-QoL instrument.

## Methodology

### RP patient survey

Given the impracticability to perform multilingual qualitative semi-structured interviews in a very rare disease such as RP, the steering committee, composed of 9 physicians, one clinical psychologist, and 2 patient representatives, prepared a script (cf. supplementary material 1) for an online structured survey. The aim of this survey was to elicit the impact of the disease and of its treatments upon the life of RP patients as closely as what could be obtained through semi-structured interviews [11]. The script comprised open-ended questions and was discussed iteratively within the steering committee with the help of the clinical psychologist (S. S.-R.) upon consensual approval. In a first section of the survey, RP patients were asked to spontaneously report facets of their lives impacted by RP. In a second section of the survey, patients were asked to provide details regarding potential RP impact upon their life, across the facets and domains of the World Health Organization Quality of Life (WHO-QoL) 100 instrument [12]. Finally, participants were asked to provide details regarding the impact of RP upon additional facets not captured by those of the WHO-QoL 100. Following cross-cultural adaptation (cf. below) the survey was disseminated online using social media (Twitter/X, Facebook) and direct contact with several RP patient associations. All participants gave informed consent, and the study was approved by the Ethic Review Board of Strasbourg University (CE-2024-15).

This study was designed with the help of an expert in qualitative research (H.D.) and used a structured online survey with open-ended questions, generating qualitative textual data. Responses were analyzed using thematic analysis. Two independent native-speaking coders per language conducted the initial coding, followed by consensus discussions and harmonization across language groups. The analytical process was supervised by a clinical psychologist experienced in qualitative research.

### Cross-cultural survey script adaptation

The survey script survey was cross-culturally adapted from English to French, Italian, Romanian, German and Portuguese, using the ERN ReCONNET methodology for cross-cultural adaptation of CTD documents in European languages. Briefly, the key-terms (major meaningful elements of the script) were identified by the steering committee, and independently assessed for their existence and similar interpretation in each of the target languages, by three translators for each target language. Any key-term reported as cross-culturally different, was first discussed within the reporting language group for confirmation, and if the discrepancy was confirmed, escalated to all participants from all language groups, asking to verify whether that specific key-term could also be discrepant in their language. Any discrepancy, if any, would be further resolved at that stage. Then, the script for the survey underwent three independent forward translations from English to each of the target languages. A reconciliation meeting was organized between the translators, and any discrepancy was logged and eventually resolved by consensus. Finally, a concluding meeting was organized for each target language, with the 3 translators and one RP patient, to ensure that the final document reflected the original interview script in terms of content and sense, and was suitable, relevant, clear and comprehensible by RP patients.

### Qualitative survey analysis

The survey data were analyzed using thematic analysis. For each language group, two native-speaking coders independently reviewed all free-text responses and carefully identified aspects related to the impact of RP on patients’ lives. These aspects were then grouped into meaningful categories to derive preliminary themes. Any coding discrepancies were resolved through discussion between the two coders. To enhance analytical rigor, the constant comparative method was applied throughout the process, enabling the themes to be refined as new data emerged. The most frequently mentioned topics helped identify which areas of life were most affected, while interpretation focused on the underlying meaning of these responses rather than on literal word counts. Broader conceptual patterns were then grouped into thematic categories reflecting patients’ lived experiences. Final harmonization across languages was performed by the central research team to ensure consistency and conceptual coherence.

The emerging themes were then re-evaluated by two steering committee members (P.M. and L.A.), who conducted a cross-language synthesis to confirm their validity. To quantitatively interpret the qualitative data, we extracted all keywords mentioned by at least five participants in English, two in French and German, and one in Romanian and Italian. Where necessary, these keywords were backtranslated into English and each was independently assessed by the same two authors for its relevance in representing the burden of RP. Any disagreements were resolved through consensus after re-examination of the original data. The selected keywords were then grouped by thematic category and reported alongside the number of patients who mentioned them. In cases of lexical variation around a core concept, occurrences were consolidated under a unified label.

### Statistical analysis

Descriptive statistics included numbers and percentages, as well as median values and 25th-75th interquartile range (IQR). Interaction with participants was conducted by email and using the Limesurvey online platform. Statistical analysis was performed using the software JMP v13.0 (SAS Institute, USA).

## Results

A total of 274 RP patients (86% women) with a median age of 54 years (IQR: 45–61) from 22 different countries completed the questionnaire (cf. supplementary material 2). Most participants were from the USA (29.9%), France (17.9%) and the UK (14.6%).

### Facets self-reported by RP patients

The first question of the survey allowed patients to spontaneously self-report the main facets of their lives affected by RP. The median number of facets affected per patient was 3 (IQR25-75: 2–6). The top three affected facets were “Personal relationships” (58.8%), “Work capacity” (51.8%) and “Activities of Daily Living” (47.8%). All affected facets are presented in Table 1.

**Table 1:** Facets of quality of life affected by RP (WHOQOL-100 framework)

Facets	*n* (%)
Personal relationships	161 (58.8)
Work capacity	142 (51.8)
Activities of daily living	131 (47.8)
Participation in and opportunities for recreation/ leisure activities	94 (34.3)
Sleep and rest	92 (33.6)
Energy and fatigue	72 (26.3)
Mobility	68 (24.8)
Others	67 (24.45)
Pain and discomfort	66 (24.1)
Negative feelings	55 (20.1)
Dependence on medication or treatments	48 (17.5)
Physical environnement	40 (14.6)
Home environment	27 (9.85)
Transport	20 (7.3)
Thinking, learning, memory and concentration	19 (6.9)
Financial resources	17 (6.2)
Bodily image and appearance	16 (5.8)
Health and social care- accessibility and quality	14 (5.1)
Sexual activity	13 (4.7)
Social support	11 (4)
Self esteem	6 (2.2)
Opportunities for acquiring new information and skills	3 (1.1)
Spirituality	2 (0.7)
Physical safety and security	1 (0.4)

### Detailed RP impact upon major HR-QoL facets

The burden of RP was evident through most HR-QoL captured by generic QoL instruments (results are presented in a Table in the supplementary material 3).

#### Energy and fatigue

‘Sleep’ disturbances (reported by 170 patients) including difficulties to ‘fall asleep’ (*n* = 10) or ‘insomnia’ (*n* = 7), particularly due to pain (*cf. below*) were highly prevalent, and associated with a marked reduction in ‘energy’ levels (*n* = 130). Patients reported being ‘tired’ (*n* = 71) or suffering from ‘fatigue’ (*n* = 64). Some of them reported being ‘exhausted’ (*n* = 26), with the need to ‘rest’ (*n* = 34) or ‘take a nap’ (*n* = 6) during the day.

#### Pain and physical discomfort

‘Pain’ (*n* = 119) was a major concern of RP patients, is occasionally reported as ‘chronic’ (*n* = 20), and in multiple body parts including the ‘ears’ (*n* = 58), the ‘nose’ (*n* = 45), the ‘joints’ (*n* = 34), the ‘knees’ (*n* = 15), the ‘feet’ (*n* = 14), the ‘hands’ (*n* = 12), the ‘cartilage’ (*n* = 12), the ‘ribs’ (*n* = 10), as ‘headaches’ (*n* = 22), in the ‘muscles’ (*n* = 8), ‘ankles’ (*n* = 7), ‘wrists’ (*n* = 6), ‘bones’ (*n* = 5), ‘thorax’ (*n* = 2) and the ‘neck’ (*n* = 2). Issues related to ‘ache’ (*n* = 16), ‘sore’ (*n* = 16) or ‘swollen’ (*n* = 9) joints or ‘arthritis’ (*n* = 7) and ‘costochondritis’ (*n* = 6) are also specifically reported. Patients describe their symptoms as ‘painful’ (*n* = 34) and responsible for ‘limited movements’ (n = 2), resulting in ‘disability’ (n = 18) with the need to use a ‘wheelchair’ (n = 5). ‘Physical’ (n = 34) symptoms and ‘discomfort’ (n = 5) impact the daily functioning.

#### Work and daily activities

‘Work’ (*n* = 132) and ‘daily tasks’ (*n* = 57) were adversely affected by the disease. Issues related to ‘job’ (*n* = 27) responsibilities were common. ‘Professional’ (*n* = 21) life and ‘career’ (*n* = 14) progression were hindered. The overall impact on ‘employment’ status (*n* = 5) and job security appeared significant.

Additionally, some patients reported difficulties in ‘learning’ (*n* = 25) or ‘studying’ (*n* = 10), with some patients dropping out of university (*n* = 5) because of limited ‘learning capacities’ (n = 25) due to the disease.

Patients also reported ‘difficulties’ (*n* = 73) in engaging into ‘physical activities’ (n = 63), ‘exercise’ (n = 24) due to their illness (n = 25). Specific mentions include issues to ‘walk’ (n = 23), ‘run’ (n = 10) or ‘drive’ (n = 8).

Altogether, reduction in energy levels, alongside with fatigue and pain were responsible for a diminution in the capacity of ‘being able’ (*n* = 61) / ‘unable’ (*n* = 36) to perform daily tasks or other ‘abilities’ (n = 25), everything seeming more ‘hard’ (n = 51). Moreover, several participants reported being ‘less active’ (n = 24) due to all of the above.

For some participants, managing ‘home’ (*n* = 35) or the ‘house’ (*n* = 14) / ‘household’ (*n* = 11) became difficult, with specific household ‘chores’ (n = 5) such as ‘cleaning’ (n = 9) being harder to perform. ‘Cooking’ meals (n = 7) became a challenging task, and other physiological activities such as ‘going to the bathroom’ (n = 5) were also impacted.

#### Specific RP symptoms & sensory organ involvement

Patients reported diverse RP ‘symptoms’ (*n* = 48) related to respiratory-tract manifestations involving the ‘trachea’ (*n* = 12), ‘airway’ (*n* = 5), ‘windpipe’ (*n* = 3), and ‘larynx’ (*n* = 2), such as ‘breathing’ (*n* = 23) or ‘talking’ (*n* = 18) difficulties, ‘voice’ (*n* = 12) changes and ‘hoarseness’ (*n* = 2), ‘shortness’ of breath (n = 6) and ‘coughing’ (n = 5).

Other specific health issues reported include ‘hearing’ loss (*n* = 22) and ‘auditory’ issues (*n* = 2), ‘tinnitus’ (n = 7), as well as ‘vision’ problems (n = 8) due to ‘eye’ involvement (n = 31). ‘Nose’ (n = 45) issues were also prevalent, alongside problems related to ‘skin’ (n = 28), ‘heart’ (n = 8) and ‘vestibular’ (n = 2) involvement. Altogether, the fear for the occurrence of ‘damage’ (n = 23) related to the disease was highlighted. 

#### Cognitive and mental health

Impaired ‘brain’ (n=54) and ‘mind’ (n=18) functioning, leading to ‘mental‘ strain (n=16) and ‘cognitive’ difficulties (n=11) were frequently reported by patients. Impaired ability to ‘think’ (n=71) or ‘concentrate’ (n=53) with ‘memory’ (n=69) and ‘brain fog’ (n=43) issues, decreased ability to ‘focus’ (n=24) and a tendency to ‘forget’ (n=11) with episodes of ‘dizziness’ (n=3), were commonly reported. Additionally, ‘mental’ (n=16) (or ‘psychic’ [n=4]) health ‘challenges’ (n=6) were specifically reported by RP patients, and include feeling ‘stress(ed)’ (n=35) [‘stressful’ (n=9)], ‘depression’ (n=20), ‘anxiety’ (n=14) [being ‘anxious’ (n=9)], with ‘mood’ instability (n=5), and overall being ‘psychologically’ impacted (n=4) and ‘morally’ not well (n=2).

#### Negative and positive feelings

Patients expressed a wide range of emotions in relation to their condition. Negative feelings such as feeling ‘hurt’ (*n* = 16) or the feeling of ‘frustration’ (*n* = 11) or ‘worry’ (*n* = 10), particularly for their ‘health’ (*n* = 37) were commonly reported. Patients reported a lack of ‘self-esteem’ (*n* = 21) or ‘confidence’ (*n* = 11) with decreased ‘motivation’ (*n* = 5) to carry out daily tasks and face the ‘challenges’ (n = 6) posed by this ‘debilitating’ condition (n = 11). They sometimes feel that their social environment ‘does not understand’ them (n = 48).

Some patients also reported an impact in ‘general desire’ (*n* = 6), but also in specific aspects such as ‘desire for children’ (*n* = 2).

On the other hand, a few patients reported trying to maintain an ‘optimistic’ outlook (*n* = 2) despite the difficulties. Descriptions of what is ‘beneficial’ (*n* = 2) in managing the disease highlight the importance of positive support and coping strategies, but this was mentioned by very few participants.

#### Social and family life

‘Family’ dynamics (*n* = 102) and ‘relationships’ (*n* = 26) were significantly impacted by RP. Specific issues arose in relationships with ‘husbands’ (*n* = 42), ‘spouses’ (*n* = 4), ‘partners’ (n = 13), ‘couple’ (n = 5) and ‘girlfriend’ (n = 2), highlighting the strain on personal relationships. Some patients reported difficulties for being ‘intimate’ with their partners (n = 22).

Being a ‘parent’ (*n* = 7) was also impacted, and responsibilities and interactions with ‘children’ (*n* = 25), ‘daughters’ (*n* = 8) and ‘kids’ (n = 13) may become challenging. Some parents had difficulties bringing their children to ‘school’ (n = 12).

‘Social’ (*n* = 22) life and interactions were broadly impacted, affecting relationships with ‘friends’ (*n* = 85), ‘others’ (*n* = 36), and ‘colleagues’ (n = 3). The need for ‘empathy’ (n = 12) and understanding from others was highlighted by many patients. They reported difficulty to have a ‘normal life’ (n = 38).

In some cases, the need for a specific ‘diet’ (*n* = 5) [for instance, along with glucocorticoid use] may also have impacted social relationships.

There was a notable need for ‘support’ (*n* = 33) from family and friends, and the necessity of ‘help’ (*n* = 55) from others was frequently mentioned. Patients described what types of assistance were ‘helpful’ (*n* = 16) and note their increased ‘dependency’ (*n* = 5) on others for daily tasks.

#### Body image

RP ‘impacts’ (n = 78) ‘body’ (n = 83) appearance (n = 7) and ‘image’ (n = 2). Some patients feared that some parts of their body will be ‘atrophying’ (n = 2). Many reported an increase in ‘weight’ (n = 42) or the fear of ‘gaining’ (n = 27) weight or ‘getting fat’ (n = 6) or ‘overweighed’ (n = 5). Loss of ‘hair’ (n = 22) was also reported.

#### Leisure and recreational activities

Engaging in leisure and recreational activities became challenging for many RP patients. Participating in ‘sports’ (*n* = 12), ‘travel’ (*n* = 15), ‘trips’ (n = 5) or going on ‘vacation’ (n = 2) was often impacted by the disease. Other activities such as ‘reading’ (n = 10) a ‘book’ (n = 2), ‘gardening’ (n = 6), and ‘biking’ (n = 2) were also affected.

#### Medical management of RP

Patients reported frequent interactions with ‘doctors’ (*n* = 44), specifically mentioning ‘rheumatologists’ (*n* = 12). They feared that limited ‘knowledge’ (*n* = 18) of physicians about RP might limit the optimal management of the disease and the way they ‘treat’ the disease (*n* = 5). The burden of ‘hospital’ (*n* = 10) visits and stays were reported, along with the need for medical ‘appointments’ (n = 16) and ‘blood’ tests (n = 16) to detect ‘inflammation’ (n = 23).

The use of various ‘medications’ (*n* = 74) and their impacts was an important concern for RP patients. Different ‘treatments’ (*n* = 41) or general ‘drugs’ [used for ‘medications’] (*n* = 13) and their efficacy were frequently discussed, with some patients experiencing ‘side effects’ (*n* = 8) including ‘infections’ (*n* = 12) or ‘diabetes’ (*n* = 6). Specific treatments mentioned include ‘infusions’ (*n* = 7), ‘injections’ (n = 5), and ‘biologic’ (n = 5) treatments. ‘Steroid’ (n = 37) therapy is frequently reported, mainly ‘prednisone’ (n = 22), ‘cortisone’ (n = 19) and ‘prednisolone’ (n = 7). Other treatments mentioned were ‘Methotrexate’ (n = 28) [sometimes termed ‘Mtx’ (n = 10)], ‘cellcept’ (n = 3) and ‘humira’ (n = 5).

#### Financial impact

Some participants reported that RP imposed a significant burden for managing ‘money’ (*n* = 6) and maintaining a steady ‘income’ (*n* = 6). The cost of medical care and treatments was described as ‘expensive’ (*n* = 5) by some participants.

#### Daily organization and future planning

Patients struggled with daily organization and future planning. Making ‘plans’ (*n* = 22) for the ‘future’ (*n* = 22) and managing a ‘daily schedule’ (*n* = 6) might become difficult. RP was described as ‘unpredictable’ (*n* = 12), and patients often tried to avoid ‘flares’ (*n* = 41), ‘crisis’ (*n* = 7), ‘episodes’ (*n* = 7) or ‘attacks’ (n = 6) with various coping mechanisms.

### Additional disease-specific QoL facets

A total of 67 patients (24.5%) reported 20 keywords not entirely or correctly captured by the WHO-QOL 100 instrument. The main concerns were related to difficulties to ‘plan’ or ‘organize’ the day (*n* = 14) and specific issues regarding ‘verbal communication’ (*n* = 13), ‘vision impairment’ (*n* = 10) and ‘smell’ impairment (*n* = 1). Avoiding flare ‘triggers’ (*n* = 8) was also a major concern, as well as not being ‘taken seriously’ by those around them (*n* = 7). Patients also reported suffering from medication ‘side effects’ (*n* = 7), ‘stress’ (*n* = 5) and ‘anxiety’ (*n* = 4). Additional themes were ‘hearing impairment’ (*n* = 3), ‘food restrictions’ (*n* = 3), ‘fear’ and ‘panic’ (*n* = 3), concerns about the ‘future’ (1), concerns about ‘life expectancy’ (*n* = 1), ‘holiday’ management (*n* = 1), ‘blood donation’ (*n* = 1), ‘lack of knowledge’ of the disease from healthcare providers (*n* = 1), RP impacted on the ‘medical management of other diseases’ (n = 1), ‘life’ (n = 1), ‘family planning’ (n = 1).

To provide greater transparency into the analytic reasoning, representative patient quotes were paired with the authors’ interpretation of their underlying meaning.

These excerpts illustrate how the analysis moved beyond word frequency to identify broader conceptual themes reflecting patients’ lived experiences across physical, psychological, and social dimensions. The complete set of illustrative excerpts and thematic interpretations is available in Supplementary Table 4. These qualitative themes formed the conceptual foundation for the subsequent development of the RP-QoL instrument and demonstrate the value of interpretative analysis in capturing the depth of patients’ lived experiences.

## Discussion

Our study emphasizes the chronic and debilitating nature of RP and its capacity to disrupt nearly every aspect of a patient’s life.

To the best of our knowledge, this is the first study to assess QoL in RP. The involvement of 274 patients from 22 countries enabled the identification of several key areas that were significantly affected by RP among a large and diverse patient sample. The facets most frequently impacted were those relating to personal relationships, work capacity and daily activities. These findings demonstrate the significant social and functional impairments experienced by RP patients, which frequently result in social isolation, reduced productivity, and difficulties in maintaining a normal life. This burden may be amplified by hearing and visual sensory impairments which may be imperfectly captured by generic HR-QoL instruments.

One of the most striking findings is the high prevalence of challenges in personal relationships, with ≈ 60% of participants reporting significant difficulties in this area. This is linked to a considerable social and emotional impact and is likely due to both the physical limitations imposed by the disease as well as to the psychological burden of living with this chronic condition. The impact of the disease on social and family life has the potential to strain relationships, particularly in the context of marital and parental roles. This can result in an increased dependency on others for daily tasks. This dimension further intensifies the psychological burden on patients, who frequently report a sense of being misunderstood or unsupported by their social environment.

Similarly, the significant effects on work capacity (51.8%) and daily activities (47.8%) further illustrate how RP can severely disrupt patients’ productivity and financial autonomy, with notable socioeconomic implications which had not been well-identified before this study.

The survey also revealed that fatigue and pain are particularly prevalent and burdensome symptoms. Importantly, pain was highly related to chondritis, especially in the ears and the nose. Also, changes in physical appearance due to deformities have a major impact on the way people perceive themselves. Both symptoms not only contribute to physical discomfort but also exacerbate psychological distress, as evidenced by the high rates of mental health challenges, including stress, depression and anxiety.

Importantly, current generic QoL instruments may prove inadequate in addressing the distinctive challenges confronted by RP patients, including the burden of chondritis, fear of deformities, unpredictability of disease progression, difficulties in verbal communication, and specific sensory impairments. Altogether, these findings further highlight the necessity for developing and validating a disease-specific HR-QoL instrument for RP, such as the RP-QoL, as this would more accurately reflect the lived experiences of RP patients.

Previous research into autoimmune diseases has shown that generic HR-QoL tools often fail to capture disease-specific burdens. In the case of RA, for instance, specialized instruments such as the RA-QoL Questionnaire [6] or the RAQoL Scale [7] have been developed to increase sensitivity to factors such as fatigue, functional limitations, and fluctuating disease activity — dimensions that generic questionnaires do not fully address. Similar advances have been made in systemic lupus erythematosus (the LupusQoL tool [13]) and ANCA-associated vasculitis (the AAV-PRO tool [14]), for which bespoke tools were designed to reflect the unique physical, emotional, and social challenges of these conditions. Our study supports this approach: many concerns expressed by RP patients — including fluctuating flare patterns, airway involvement, and a lack of societal or medical understanding — are not adequately covered by the WHOQoL-100. These findings reinforce the necessity of a disease-specific instrument for RP that can capture the full complexity of patients’ lived experiences.

Importantly, current generic QoL instruments may prove inadequate in addressing the distinctive challenges confronted by RP patients, including the burden of chondritis, fear of deformities, unpredictability of disease progression, difficulties in verbal communication, and specific sensory impairments. Altogether, these findings further highlight the necessity for developing and validating a disease-specific HR-QoL instrument for RP, such as the RP-QoL, as this would more accurately reflect the lived experiences of RP patients.

While this study provides valuable insights into the impact of RP on HR-QoL, it is important to acknowledge some limitations inherent to its design, including the qualitative nature of the study. First, due to the rarity of RP and the potential for sensory impairments such as hearing loss, conducting multilingual qualitative interviews was not feasible. As a pragmatic alternative, a structured online survey was developed with input from a qualitative research expert to ensure methodological robustness despite the inherent limitations in response depth.

Another limitation resides in the fact that the majority of participants originated from high-income countries, namely the USA, France, Germany, Italy and the UK, where the burden of the disease may differ from that of those from low and middle-income countries (LMICs). However, the inclusion of patients from 22 distinct countries, including some LMICs, may mitigate this issue. Secondly, the utilization of an online structured survey may have resulted in the exclusion of certain patient groups who lack internet access or are reluctant to engage in digital communication. Conversely, this approach has permitted a global distribution of the study, with the participation of a large number of patients worldwide in a very rare disease. Of note, this innovative methodology has already been validated in previous studies [11, 15, 16]. The next limitation relates to the confirmation of diagnosis in an anonymous online context. However, it is noteworthy that the questionnaire explicitly sought confirmation of the respondents’ diagnosis of RP.

Additionally, the initial survey script was developed by physicians and a clinical psychologist, in collaboration with patient representatives. While other healthcare professionals (e.g. nurses and occupational therapists) were not involved at this stage, the development of future phases of the RP-QoL tool will incorporate input from a broader range of disciplines to refine and validate the identified facets.

Previous research into autoimmune diseases has shown that generic health-related quality of life (HR-QoL) tools often fail to capture disease-specific burdens. In the case of rheumatoid arthritis (RA), for example, the development of specialised instruments such as the RAQoL and RAQoL-S has increased sensitivity to factors such as fatigue, functional limitations and fluctuating disease activity — dimensions that are not fully addressed by generic questionnaires. Similar advances have been made in systemic lupus erythematosus (the LupusQoL tool) and ANCA-associated vasculitis (the AAV-PRO tool), where bespoke tools were designed to reflect the unique physical, emotional and social challenges of these conditions. Our study supports this approach; many concerns expressed by RP patients, including fluctuating flare patterns, airway involvement and a lack of societal or medical understanding, are not adequately covered by the WHOQoL-100. These findings reinforce the necessity of a disease-specific instrument for RP that is capable of capturing the full complexity of patients’ lived experiences.

Our study reveals the profound and multifaceted impact of RP on patients’ lives, emphasizing its chronic and debilitating nature. This comprehensive analysis demonstrates how the disease disrupts nearly every aspect of daily functioning, from intimate relationships to professional capabilities.

To our knowledge, this represents the first large-scale assessment of quality of life in RP patients. By engaging 274 participants across 22 countries, we have identified critical areas of impairment that affect this diverse patient population. The most severely impacted domains consistently involved personal relationships, work capacity, and daily activities—findings that illuminate the extensive social and functional challenges faced by individuals living with RP. These impairments frequently culminate in social isolation, diminished productivity, and profound difficulties maintaining normalcy in daily life. The burden appears particularly pronounced given the potential for concurrent hearing and visual sensory deficits, which may not be adequately captured by conventional generic HR-QoL instruments.

Perhaps our most striking finding is the overwhelming prevalence of relationship difficulties, affecting approximately 60% of participants. This substantial impact reflects both the physical constraints imposed by the disease and the psychological burden of managing a chronic, unpredictable condition. The ripple effects extend through social and family networks, potentially straining marriages and parental relationships while increasing dependency on others for routine tasks. This social dimension intensifies the psychological burden, as patients frequently describe feeling misunderstood or inadequately supported by their environment. The isolation becomes self-perpetuating, as physical limitations and emotional distress compound to further restrict social engagement.

The significant impact on work capacity (51.8%) and daily activities (47.8%) demonstrates how RP severely compromises patients’ productivity and financial independence. These findings reveal previously under-recognized socioeconomic implications, highlighting the broader societal burden of this rare disease.

Our survey highlighted the particularly challenging impact of fatigue and pain on the lives of RP patients. Pain was strongly associated with chondritis, particularly affecting the ears and nose, and physical deformities significantly impacted patients’ self-perception and body image. These symptoms create a vicious cycle where physical discomfort exacerbates psychological distress, resulting in elevated stress, depression and anxiety levels.

Current generic HR-QoL instruments appear to be inadequate for capturing the unique challenges faced by RP patients. The distinctive burden of chondritis, the fear of progressive deformities, unpredictable disease trajectories, communication difficulties and specific sensory impairments require a more nuanced approach to assessment. These findings strongly support the development and validation of disease-specific instruments, such as the RP-QoL, which would provide a more accurate reflection of patients’ lived experiences.

There is a well-established framework for disease-specific tools in autoimmune conditions. In the case of RA, specialised instruments such as the RAQoL [6] and RAQoL-S [7] have increased sensitivity to disease-specific factors, including fatigue, functional limitations and fluctuating activity. These dimensions are inadequately addressed by generic questionnaires. Similar advances have been made in the assessment of systemic lupus erythematosus (LupusQoL [13]) and ANCA-associated vasculitis (AAV-PRO [14]), demonstrating the value of tools designed specifically to capture the unique physical, emotional and social challenges of these conditions. Our findings reinforce this approach, as many RP-specific concerns, including fluctuating flare patterns, airway involvement and societal misunderstanding, remain poorly captured by instruments such as the WHOQoL-100. A disease-specific tool could reflect the complexity of patients’ experiences with RP more comprehensively.

Although our methodology enabled unprecedented global participation in RP research, there are certain limitations that merit acknowledgement. Without prompts or follow-up questions, researchers could not clarify participants’ intended meanings or explore emerging themes in more depth. This limitation was acknowledged during the analysis stage and addressed through multiple rounds of discussion between coders and cross-language triangulation, ensuring interpretative rigor and conceptual coherence. Despite these constraints, the structured online format enabled unprecedentedly broad participation in the context of this ultra-rare disease.

Using structured textual responses rather than traditional qualitative interviews inherently limits narrative richness and spontaneous elaboration. Consequently, subtle emotional expressions and unexpected themes may be underrepresented. Nevertheless, the substantial dataset remains highly informative for future interview-based qualitative research. The rarity of RP and the potential for sensory impairments meant that conducting multilingual qualitative interviews was impractical. Our structured online survey, developed with the help of qualitative research experts, is a pragmatic alternative that maintains methodological rigor despite the limitations in terms of depth.

Another consideration is geographic representation, as most participants originated from high-income countries, including the USA, France, Germany, Italy and the UK. The disease burden may differ in low- and middle-income countries (LMICs), though including patients from 22 countries, including some LMICs, provides valuable diversity. However, the online format may have inadvertently excluded patients who lack internet access or are not comfortable with digital communication. Nevertheless, this approach enabled us to reach people around the world with a very rare disease, utilizing methodology that has been validated in similar studies. Obtaining diagnostic confirmation in an anonymous online context posed challenges, although our questionnaire explicitly sought confirmation of RP diagnosis from respondents.

Finally, while our initial RP-QoL tool development involved physicians, clinical psychologists and patient representatives, future development will incorporate broader healthcare perspectives, including nurses and occupational therapists, to refine and validate the identified domains.

This study establishes a foundation for understanding the impact of RP on quality of life, while also highlighting the urgent need for disease-specific assessment tools. The comprehensive impairment across personal, professional and functional domains highlights the need for a holistic approach to care that addresses not only the physical manifestations of RP, but also its profound psychosocial consequences.

## Conclusions

The present study provides valuable insights into the burden of RP, as well as the multiple ways by which RP affects patients’ HR-QoL. This further highlights the need for individualized approaches to healthcare. Importantly, our results allowed the identification of disease-specific manifestations that are not adequately addressed by generic HR-QoL instruments. These include pain related to chondritis and changes in physical appearance due to deformities. Additionally, hearing and visual sensory impairment were also identified. Altogether, these data further support the need for the development of a disease-specific HR-QoL instrument, the RP-QoL. Once developed from the facets identified above, and validated, the RP-QoL will enable a more comprehensive understanding of the impact of RP on patients’ lives.

## Supplementary Information

Below is the link to the electronic supplementary material.

Supplementary Material 1

## Data Availability

All data relevant to the study are included in the article.

## Declarations

### Disclaimer

The content of this publication represents the views of the authors only and it is their sole responsibility; it cannot be considered to reflect the views of the European Commission and/or the Health and Digital Executive Agency (HaDEA) or any other body of the European Union. The European Commission and the Agency do not accept any responsibility for use that may be made of the information it contains.

## Abbreviations

RP: Relapsing polychondritis
QoL: Quality of life
HR-QoL: Health-related Quality of life
ERN ReCONNET: European Reference Network on Connective Tissue and Musculoskeletal Diseases
WHO-QoL: World Health Organization Quality of Life
IQR: Interquartile range
LMICs: Low and middle-income countries
