# Supplementary material for: Identifying quality of life domains and facets affected in relapsing polychondritis: a qualitative analysis for the development of a disease-specific health-related quality of life instrument
Source: Orphanet J Rare Dis. 2026 Mar 11;21:157. doi: 10.1186/s13023-026-04297-3 (PMC13088433; doi:10.1186/s13023-026-04297-3)
Supplement: Supplementary file 1 — Supplementary Material 1 [file 13023_2026_4297_MOESM1_ESM.docx]

**SUPPLEMENTARY MATERIAL**

**Supplementary material 1: Script for interview survey**

**Thank you for agreeing to participate in this research about quality of life in Relapsing Polychondritis.**

This survey is designed as an “online interview”, to better understand what persons living with relapsing polychondritis (RP) feel, and how RP affects their daily life. So there are no right or wrong answers to any of our questions, we are truly interested in *your own* *experiences.*

Participation in this study is voluntary and your decision to participate, or not participate, will not affect the care you currently receive from your usual medical team.

**This survey should take approximately 30-50 minutes, depending on how much information you would like to share. We suggest that you start only when you have this amount of time available.**

All your responses will be kept strictly confidential. This means that your responses are collected using an anonymous survey system (*no computer IP recorded*) and will only be shared with research team. We will ensure at all time that any information we include in our report does not identify you as the respondent. Besides, you may decline to answer any question or stop the survey at any time and for any reason.

| Question A1 | **Please list the areas of your life (considering all aspects) that are impacted by relapsing polychondritis**   - *Feel free to mention as many elements as you wish* - *There is no need to provide details at that stage, as we will ask you more specific questions during the remaining of the interview* - *Please note that you will not be able to come back to this question once you have clicked ‘next’.* |
| --- | --- |
| **Now we would like to understand more precisely the impact of relapsing polychondritis upon several domains of life**   - *Please note that this section contains a total of 10 questions (so that you can manage your time!)* - *When answering, please try to explain in your own words what is the impact of relapsing polychondritis upon the domain considered, and how it affects your life* - ***When possible, try to avoid very short answers such as: ‘yes’, ‘no’, ‘no impact’, or ‘a lot’*** | |
| Question B1 | How would you describe the impact of relapsing polychondritis on your **daily life and activities**? |
| Question B2 | How would you describe the impact of relapsing polychondritis on your **professional life** (**or studies**, if you are a student)? |
| Question B3 | How would you describe the impact of RP on **relationships (family, intimate life, friends, etc…)** |
| Question B4 | How would you describe the impact of relapsing polychondritis on your **body**? |
| Question B5 | How would you describe the impact of relapsing polychondritis on the way **you see yourself?** |
| Question B6 | How would you describe the impact of relapsing polychondritis **on your mind/cognition** (e.g., thinking, learning, memory and concentration, etc…) |
| Question B7 | How would you describe the impact of relapsing polychondritis on your **energy and sleep**? |
| Question B8 | How would you describe the impact of **medications and non-pharmacological treatments** for relapsing polychondritis upon your life? |
| Question B9 | Despite the impacts described in previous questions, did you identify any **positive impact** of relapsing polychondritis upon your life (e.g., learnings or personal growth)? Can you describe it? |
| Question B10 | **Is there anything else that you would like to comment on that we have not already asked you about?** |

Thank you so much for your time! Your willingness to share your experiences will benefits the whole community of persons living with relapsing polychondritis.

**Supplementary material 2: Origin of participants**

| Country | Number of patients | Spoken languages | | | | |
| --- | --- | --- | --- | --- | --- | --- |
|  |  | English | French | German | Romanian | Italian |
| United States | 82 (29.9) | 82 (100) |  |  |  |  |
| France | 49 (17.9) | 1 (2) | 48 (98) |  |  |  |
| United Kingdom | 40 (14.6) | 40 (100) |  |  |  |  |
| Germany | 23 (8.4) |  |  | 23 (100) |  |  |
| Romania | 20 (7.3) | 2 (10) |  |  | 18 (90) |  |
| Canada | 14 (5.1) | 13 (93) | 1 (7) |  |  |  |
| Australia | 10 (3.6) | 1 (100) |  |  |  |  |
| New Zealand | 8 (2.9) | 8 (100) |  |  |  |  |
| Italy | 7 (2.6) |  |  |  |  | 7 (100) |
| Switzerland | 6 (2.2) |  | 3 (50) | 3 (50) |  |  |
| South Africa | 2 (0.7) | 2 (100) |  |  |  |  |
| Sweden | 2 (0.7) | 2 (100) |  |  |  |  |
| The Netherlands | 2 (0.7) | 2 (100) |  |  |  |  |
| Argentina | 1 (0.4) | 1 (100) |  |  |  |  |
| Austria | 1 (0.4) |  |  | 1 (100) |  |  |
| Belgium | 1 (0.4) |  | 1 (100) |  |  |  |
| Bermuda | 1 (0.4) | 1 (100) |  |  |  |  |
| Luxembourg | 1 (0.4) |  |  | 1 (100) |  |  |
| Mexico | 1 (0.4) | 1 (100) |  |  |  |  |
| New Caledonia | 1 (0.4) |  | 1 (100) |  |  |  |
| Puerto Rico | 1 (0.4) | 1 (100) |  |  |  |  |
| United Arab Emirates | 1 (0.4) |  | 1 (100) |  |  |  |

Results are presented as N (%).

**Supplementary material 3: Main reported patient-terms by WHO-QoL facets and additional facets**

| Energy and fatigue | | | | | |
| --- | --- | --- | --- | --- | --- |
| Sleep (170)  Energy (130) | Tired (71)  Fatigue (64) | | Rest (34)  Exhausted (26) | | Take a nap (6) |
| Pain and physical discomfort | | | | | |
| Pain (119)  Joints (34)  Physical (34)  Ears (58)  Nose (45)  Painful (34)  Chronic (20) | headaches (22)  Disability (18)  Disability (18)  Sore (16)  feet (14)  hands (12)  cartilage (12) | | ribs (10)  swollen (9)  muscles (8)  ankles (7)  joints or arthritis (7) | | costochondritis (6)  wrists (6)  bones (5)  wheelchair (5)  discomfort (5) |
| Work and daily activities | | | | | |
| Work (132)  Difficulties (73)  Physical activities (63)  Being able (61)  Daily tasks (57)  Hard (51)  Unable (36) | Home (35)  Job (27)  Learning (25)  Learning capacities (25)  Abilities (25)  Exercise (24)  Less active (24) | | Walk (23)  Professional (21)  Career (14)  House (14)  Household (11)  Studying (10) | | Cleaning (9)  Drive (8)  Cooking (7)  Employment (5)  Chores (5)  Going to the bathroom (5) |
| Specific RP symptoms & sensory organ involvement | | | | | |
| Symptoms (48)  Nose (45)  Eye (31)  Skin (28)  Breathing (23) | Damage (23)  Hearing (22)  Talking (18)  Trachea (12) | | Voice (12)  Tinnitus (7)  Vision (8)  Heart (8) | | Airway (5)  Coughing (5)  Shortness (6) |
| Cognitive and mental health | | | | | |
| Think (71)  Memory (69)  Brain (54)  Concentrate (53) | Brain fog (43)  Stress(ed) (35)  Focus (24)  Depression (20) | | Mind (18)  Mental (16)  Anxiety (14)  Forget (11) | | Stressful (9)  Anxious (9)  Challenges (6) |
| Negative and positive feelings | | | | | |
| Health (37)  Self-esteem (21)  Hurt (16) | Frustration (11)  Confidence (11) | | Worry (10)  Challenges (6) | | Motivation (5) |
| Social and family life | | | | | |
| Friends (85)  Help (55)  Husbands (42)  Normal life (38)  Others (36) | Support (33)  Relationships (26)  Children (25)  Social (22)  Helpful (16) | | Partners (13)  Kids (13)  School (12)  Empathy (12)  Daughters (8) | | Parent (7)  Couple (5)  Diet (5)  Dependency (5) |
| Body image | | | | | |
| Body (83)  Impacts (78) | Weight (42)  Gaining (27) | | Hair (22)  Getting fat (6) | | Overweighed (5) |
| Leisure and recreational activities | | | | | |
| Travel (15)  Sports (12)  Reading (10) | | | Gardening (6)  Trips (5) | | |
| Medical management of RP | | | | | |
| Medications (74)  Doctors (44)  Treatments (41)  Steroid (37)  Methotrexate (28)  Inflammation (23) | Prednisone (22)  Cortisone (19)  Knowledge (18)  Appointments (16)  Blood (16)  Rheumatologists (12) | | Infections (12)  Methotrexate (10)  Hospital (10)  Side effects (8)  Infusions (7)  Prednisolone (7) | | Diabetes (6)  Injections (5)  Biologic (5)  Humira (5) |
| Financial impact | | | | | |
| Money (6) | | Income (6) | | Expensive (5) | |
| Daily organization and future planning | | | | | |
| Flares (41)  Plans (22)  Future (22) | | Unpredictable (12)  Crisis (7)  Episodes (7) | | Daily schedule (6)  Attacks (6) | |
| Additional disease-specific QoL facets | | | | | |
| Verbal communication (13)  Vision impairment (10) | | Triggers (8)  Side effects (7) | | Stress (5) | |

This table reports the main (n≥5) reported patient terms.

# Supplementary material 4: Representative patient quotes and authors’ interpretation.

**These quotes were selected to illustrate major conceptual domains identified through thematic analysis. They reflect typical responses reported across languages. Themes were derived through iterative, interpretative coding rather than word frequency counts.**

| **Thematic domain** | **Representative patient quotes (illustrative)** | **Authors’ interpretation**  **(latent meaning)** |
| --- | --- | --- |
| Energy and fatigue | “I’m constantly exhausted.” / “Pain keeps me awake at night.” / “I need naps every day.” | Persistent fatigue and poor sleep are central experiences of RP, leading to loss of stamina and daily functioning. Pain-induced insomnia contributes to physical and emotional exhaustion. |
| Pain and physical discomfort | “My ears and nose are always painful.” / “Everything hurts — joints, ribs, even breathing.” | Chronic, multi-site pain—especially from chondritis—disrupts comfort, mobility, and independence. Pain shapes identity as a ‘suffering body’. |
| Work and daily activities | “I had to stop working because I can’t concentrate or move much.” / “Even small chores like cooking or cleaning feel impossible.” | RP reduces autonomy and professional participation. The disease undermines productivity and independence, affecting financial stability and self-worth. |
| Cognitive and mental health | “Brain fog makes me forget things.” / “I can’t focus or think clearly anymore.” / “Stress and anxiety are constant.” | Cognitive dysfunction (‘brain fog’) and mental distress are core invisible burdens. Unpredictable symptoms trigger anxiety and loss of confidence in one’s abilities. |
| Negative feelings and emotions | “I feel frustrated, scared, and misunderstood.” / “Sometimes I lose motivation to do anything.” | Emotional fatigue, frustration, and worry are pervasive. Patients experience lowered self-esteem and loss of control, reflecting emotional isolation. |
| Social and family life | “My friends don’t understand me.” / “My partner is tired of my illness.” / “I can’t take my kids to school anymore.” | RP strains family and social relationships. Feelings of isolation and misunderstanding amplify emotional burden. Dependence on others challenges self-image. |
| Body image and appearance | “I can’t look at myself since my nose changed.” / “I’m gaining weight because of steroids.” / “I’m losing my hair.” | Alterations in appearance and weight due to inflammation and treatment affect self-identity, social interactions, and confidence. |
| Leisure and recreation | “I used to love travelling and sports — now I can’t.” / “Even reading or gardening is tiring.” | Loss of leisure activities symbolizes the shrinking of life space. Patients mourn their previous identity and spontaneity. |
| Specific RP symptoms and sensory involvement | “I can’t hear well anymore.” / “My vision is blurry.” / “Talking or breathing is painful.” | Sensory and airway symptoms uniquely define RP’s burden. They cause fear of suffocation, communication barriers, and social withdrawal. |
| Medical management and treatment burden | “Doctors don’t know much about RP.” / “I take steroids and methotrexate — side effects are terrible.” / “I’m always in hospital for tests.” | Recurrent medical procedures, limited disease knowledge among clinicians, and treatment side effects reinforce helplessness and mistrust toward the healthcare system. |
| Financial impact | “Treatment is expensive.” / “I can’t work anymore — money is tight.” | Economic vulnerability arises from job loss and chronic treatment costs, increasing psychosocial stress. |
| Daily organisation and future planning | “I never know when a flare will hit.” / “It’s impossible to plan anything.” | Disease unpredictability disrupts planning, fostering uncertainty about the future and a sense of instability. |
| Additional RP-specific facets | “People don’t take me seriously.” / “I avoid triggers to prevent flares.” / “Speaking is exhausting.” | Unique aspects such as social invalidation, verbal communication difficulty, and sensory fatigue are not captured by generic QoL tools. |
| Coping and resilience (positive aspects) | “Despite everything, I stay optimistic.” / “The illness taught me patience and empathy.” | Some patients reframe adversity into personal growth. Acceptance and resilience coexist with chronic suffering. |
